# Differential impact of malaria control interventions on *P. falciparum* and *P. vivax* infections in young Papua New Guinean children

**DOI:** 10.1186/s12916-019-1456-9

**Published:** 2019-12-09

**Authors:** Maria Ome-Kaius, Johanna Helena Kattenberg, Sophie Zaloumis, Matthew Siba, Benson Kiniboro, Shadrach Jally, Zahra Razook, Daisy Mantila, Desmond Sui, Jason Ginny, Anna Rosanas-Urgell, Stephan Karl, Thomas Obadia, Alyssa Barry, Stephen J. Rogerson, Moses Laman, Daniel Tisch, Ingrid Felger, James W. Kazura, Ivo Mueller, Leanne J. Robinson

**Affiliations:** 1 Papua New Guinea Institute of Medical Research, Madang, Papua New Guinea; 2 Walter and Eliza Hall Institute of Medical Research, Melbourne, Australia; 3 Department of Medical Biology, University of Melbourne, Melbourne, Australia; 4 Institute of Tropical Medicine, Antwerp, Belgium; 5 Institut Pasteur, Paris, France; 6 Case Western Reserve University, Cleveland, USA; 7 Swiss Tropical and Public Health Institute, Basel, Switzerland; 8 Burnet Institute, Melbourne, Australia

**Keywords:** *P. falciparum*, *P. vivax*, Papua New Guinea, Epidemiology, Malaria control, Incidence, Prevalence

## Abstract

**Introduction:**

As malaria transmission declines, understanding the differential impact of intensified control on *Plasmodium falciparum* relative to *Plasmodium vivax* and identifying key drivers of ongoing transmission is essential to guide future interventions.

**Methods:**

Three longitudinal child cohorts were conducted in Papua New Guinea before (2006/2007), during (2008) and after scale-up of control interventions (2013). In each cohort, children aged 1–5 years were actively monitored for infection and illness. Incidence of malaria episodes, molecular force of blood-stage infections (_mol_FOB) and population-averaged prevalence of infections were compared across the cohorts to investigate the impact of intensified control in young children and the key risk factors for malaria infection and illness in 2013.

**Results:**

Between 2006 and 2008, *P. falciparum* infection prevalence, _mol_FOB, and clinical malaria episodes reduced by 47%, 59% and 69%, respectively, and a further 49%, 29% and 75% from 2008 to 2013 (prevalence 41.6% to 22.1% to 11.2%; _mol_FOB: 3.4 to 1.4 to 1.0 clones/child/year; clinical episodes incidence rate (IR) 2.6 to 0.8 to IR 0.2 episodes/child/year). *P. vivax* clinical episodes declined at rates comparable to *P. falciparum* between 2006, 2008 and 2013 (IR 2.5 to 1.1 to 0.2), while *P. vivax*_mol_FOB (2006, 9.8; 2008, 12.1) and prevalence (2006, 59.6%; 2008, 65.0%) remained high in 2008. However, in 2013, *P. vivax*_mol_FOB (1.2) and prevalence (19.7%) had also substantially declined. In 2013, 89% of *P. falciparum* and 93% of *P. vivax* infections were asymptomatic, 62% and 47%, respectively, were sub-microscopic. Area of residence was the major determinant of malaria infection and illness.

**Conclusion:**

Intensified vector control and routine case management had a differential impact on rates of *P. falciparum* and *P. vivax* infections but not clinical malaria episodes in young children. This suggests comparable reductions in new mosquito-derived infections but a delayed impact on *P. vivax* relapsing infections due to a previously acquired reservoir of hypnozoites. This demonstrates the need to strengthen implementation of *P. vivax* radical cure to maximise impact of control in co-endemic areas. The high heterogeneity of malaria in 2013 highlights the importance of surveillance and targeted interventions to accelerate towards elimination.

## Background

Intensification of malaria control measures has been associated with marked reductions in transmission and infection and illness burden in many endemic areas [1]. In the Americas [1, 2] and some parts of Asia-Pacific [3, 4], these reductions have been associated with a marked shift to the predominance of *Plasmodium vivax* as the primary source of *Plasmodium* spp. infections. In parallel, the proportion of low-density, asymptomatic infections has been observed to increase [5–8] and transmission becomes more heterogeneous [9–11].

The reasons underlying these shifts are likely to be multifactorial. A major factor for the relative increase in *P. vivax* is the poor uptake and/or adherence of anti-hypnozoite therapy [12, 13]. As a result, *P. vivax* hypnozoites are able to cause repeated bouts of blood-stage parasitaemia and are responsible for up to 80% of all *P. vivax* blood-stage infections [14]. Even in low and very low transmission settings, most *P. vivax* infections are asymptomatic [15, 16] and often of very low density [16] but almost all carry detectable gametocytaemia [6, 17, 18]. These infections are thus not detected and treated by the health systems and can sustain transmission. *P. vivax* is also considered more easily transmissible given the rapid maturation and thus early presence of its gametocytes [19] and faster development cycle in its mosquito host [20]. Lastly, it has also been observed that *P. vivax*-infected mosquitoes may be younger and more likely to bite early and outdoors [21, 22]. All of these factors may render *P. vivax* transmission less susceptible to vector control and routine case management interventions.

The highly heterogeneous nature of malaria transmission across countries, between neighbouring villages and within the same village has long been recognised [23–25] and is driven by an interplay of host, vector and environmental factors [23, 26, 27]. As transmission declines, there is a tendency for malaria infections to become increasingly clustered in high-risk populations and high-risk areas [11, 28] and it becomes more important to be able to identify these clusters since they may be responsible for sustaining transmission [11]. There is growing evidence that despite achieving overall reductions in malaria transmission through improved malaria control, infections and illness burden in many hyperendemic areas remain unaltered [29–31] and that more targeted interventions may be necessary for elimination [11].

In the early 2000s, the overall burden of malaria in Papua New Guinea (PNG) was amongst the highest in the Asia-Pacific region, albeit with intensity of transmission geographically highly variable across the country [27, 32, 33]. *Plasmodium falciparum* and *P. vivax* are the two predominant species that account for most of the burden of malaria infections and illness in PNG [32, 34].

Beginning in 2004, with the support of Global Fund to Fight AIDs, Tuberculosis and Malaria, PNG scaled up its malaria control interventions through scheduled 3-yearly nationwide distribution of long-lasting insecticide treated nets (LLINs), introduction of a test-and-treat approach and a switch to artemether-lumefantrine (AL) as first-line treatment [35, 36]. Subsequent surveys revealed a substantial decline in the overall burden of malaria [6, 33], with the nationwide infection prevalence by light microscopy (LM) declining from 11.1% in 2009 to 0.9% in 2014 [33, 37]. Entomological studies also revealed a large decline in human biting rates from 83 bites/person/night to 31 bites/person/night [37, 38]. As elsewhere, these reductions in PNG have gone hand-in-hand with an increase in the proportion of asymptomatic and sub-microscopic infections [6] and a pronounced heterogeneity of residual transmission [39]. Although the prevalence of PCR-detectable *P. vivax* infections in community surveys has not declined to the same extent as *P. falciparum* infection [6], the shift towards *P. vivax* predominance has not yet been as pronounced as in neighbouring SE Asia- and SW Pacific countries [7].

To better understand the relationship between changing transmission and the risk profile of malaria infections and disease, it is vital to gain insight into the impact that control measures have on the two main species, *P. falciparum* and *P. vivax*. Using three consecutive longitudinal child cohorts (1–5-year-old children) conducted in the same study area, prior [40], during [41] and following 5 years of intensification (2013 cohort), we investigated the impact of improved malaria control on the breadth of metrics including clinical incidence, incidence of newly acquired infections (i.e. the molecular force of blood-stage infection, _mol_FOB) [42, 43] and infection prevalence to better understand changing *P. falciparum* and *P. vivax* epidemiology in the context of rapid reductions in transmission. In order to guide continued reductions in transmission, we also investigated the key drivers of infection and illness in young children during the period of low transmission in 2013.

## Methods

### Study design and sites

**Fig. 1:**
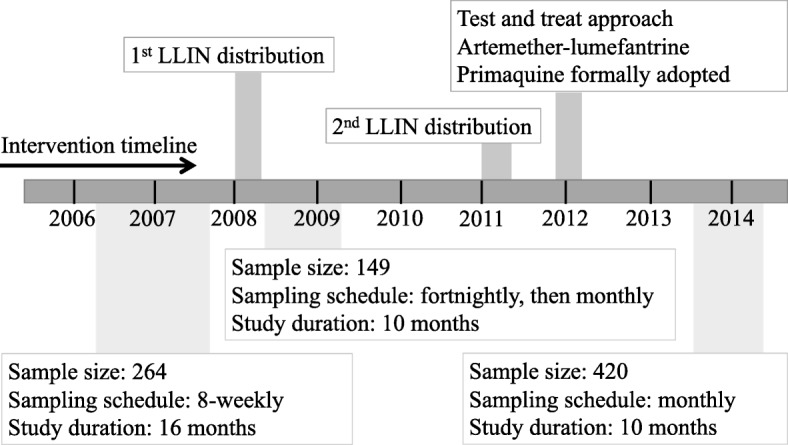
Study and intervention timeline. Legend: The timeline shows the time-points when the three cohorts were conducted in reference to malaria control interventions that occurred in the study area

Three longitudinal cohort studies of 1–5-year-old children were conducted in the same study area in the Ilahita area of Maprik District, East Sepik Province in 2006, 2008 and 2013. A detailed description of the study area is given elsewhere [40]. Briefly, the study area is located in northern PNG where malaria transmission is considered hyperendemic [34, 44] and all human malaria species are endemic [40, 41, 45, 46]. Health services are provided solely by the church-run Ilahita Health Centre with inconsistent services from a government aid post. The cohorts were conducted at three different time-points before and during the scale-up of malaria control interventions in the study area (Fig. 1).

### Cohorts

#### 2006 cohort (pre-intensification)

Children aged 1–3 years were enrolled into the study and actively followed up for malaria infection and illness every 8 weeks for a total of 16 months from March 2006 to August 2007 [40, 42, 43]. Passive case detection at Ilahita Health Centre was maintained throughout the study for detection of clinical episodes. All rapid diagnostic test (RDT) or LM confirmed febrile illness episodes were treated with AL (Coartem®, Novartis) (if treated by study staff) or amodiaquine plus sulphadoxine-pyrimethamine as per the PNG standard treatment for common illnesses in children [47] (if receiving treatment from a non-study source). Children with *P. vivax* episodes were not treated with primaquine as it had not yet been introduced into PNG standard treatment guidelines [47]. Full details of the study methodology are published [40, 42, 43].

#### 2008 cohort (during early intensification)

Children 1–5 years of age were enrolled into this randomised controlled trial in April 2008, a month after the first population-wide distribution of LLIN into the study area [41]. Analysis was restricted to the control arm to allow comparability to the other two observational studies. Children were actively checked for malaria infection and illness fortnightly for the first 3 months and monthly thereafter for another 7 months. All RDT or LM confirmed febrile illness episodes were treated with AL (Coartem®, Novartis) (if treated by study staff) or Amodiaquine plus sulphadoxine-pyrimethamine as per the PNG standard treatment guidelines [47] (if receiving treatment from a non-study source). Children with *P. vivax* episodes were not treated with primaquine as it had not yet been introduced into PNG standard treatment guidelines [47]. Full details of the study methodology are published [41].

#### 2013 cohort (5 years after sustained control)

This cohort was conducted after 5 years of sustained malaria control in the study area (Fig. 1) A total of 465 children aged 1–5 years at enrolment from 12 villages (Ilahita 1–7, Kamanokor, Sunuhu 1 and 2, Balanga and Balif) in Ilahita area were enrolled from July to September, 2013, and followed for 12 months. Of these, 45 children were excluded post hoc (11 withdrawals, 26 lost to follow-up, 8 with erratic attendance), resulting in a final sample size of 420 children (90% retention rate). All 420 children ranging in age from 0.9–6.4 years during the study period were included in the analysis investigating the key drivers of infection and illness in 2013. A subset (*n* = 371) aged ≤ 55 months were age-matched to earlier two cohorts to investigate the changing burden of malaria across the intervention time-points.

At enrolment, demographic and clinical data on recent illness and medications, bednet use and current state of health were recorded. Axillary temperatures were measured using an electronic digital thermometer. A 5-ml (ml) venous blood sample and two blood slides were collected. Haemoglobin level was measured using a portable HemoCue machine (HemoCue, Angholm, Sweden). The location of each child’s residence was recorded using a Garmin eTrex®.

Following enrolment, children were actively followed up fortnightly for morbidity surveillance and monthly for blood sampling (250 μl finger prick sample, two blood slides and haemoglobin measurement). If a child had a febrile illness at a morbidity surveillance visit, a finger prick sample of 250 μL blood and 2 blood slides were collected. RDT for malaria was performed and, if positive, children were treated with AL (Coartem®, Novartis) and occasionally AL plus primaquine for RDT positive *P. vivax*, as per PNG standard treatment guidelines [48]. Over the course of the study, 9 children were documented as receiving primaquine, suggesting that primaquine was inconsistently administered by health workers. Anaemic children with haemoglobin < 7.5 g/dL were given an anthelminthic drug (albendazole) and iron supplementations while other ailments were treated according to PNG standard treatment [48].

*Plasmodium* spp. infections were detected by real-time quantitative PCR assay (qPCR), as previously described [40–43, 49] and LM. Briefly, parasite DNA was extracted from cell pellets (equivalent to 200 μL whole blood) using a Favorgen 96-well Genomic DNA Extraction Kit following the manufacturer’s instructions and eluted in 200 μL elution buffer. The presence of *P. falciparum*, *P. vivax*, *P. malariae* and *P. ovale* infections were determined using two multiplex 2-species qPCR assays [49]. Infections with *P. falciparum* and *P. vivax* were further genotyped for *Pfmsp2*, *Pvmsp1F3* and *PvMS16* to identify individual parasite clones. All blood slides positive by first read and/or by *Plasmodium* screening qPCR [50], as well as 10% of the negatives, were independently examined by a second microscopist. Any discrepancies between the first and the second reads were then re-read by a third expert-level microscopist (WHO Level 1 certified). The final density was calculated by taking the geometric mean of the two concordant reads.

### Statistical analysis

Analysis for this paper occurred in two parts and focussed on the two predominant species, *P. falciparum* and *P. vivax*. In the first part “Analysis of changing burden of malaria infections and illness: 2006 – 2013”, we aimed to compare the prevalence, _mol_FOB and clinical incidence across the three cohorts to determine patterns of decline for *P. falciparum* relative to *P. vivax* across the intervention time-points. In the second part, “Analysis of key determinants of malaria infection and illness during the time of low transmission 2013”, the objective was to explore the full dataset of the 2013 cohort to identity factors that were key predictors of infection and illness during the period of low transmission in 2013. In both analyses, a clinical malaria episode was defined as history of febrile illness during the preceding 48 h and/or measured temperature ≥ 37.5 °C in the presence of a microscopically detectable infection of any density. The _mol_FOB (number of genetically unique blood-stage infections) was calculated from the number of new infections acquired during the intervals between sampling time-points by counting all new *msp2* alleles for *P. falciparum* and *msp1F3* and *MS16* alleles for *P. vivax* per unit time that were not present in the preceding intervals.

#### Analysis of changing burden of malaria infections and illness: 2006–2013

Data from each cohort were analysed separately due to the differences in the sampling schedules and the length of follow-up between the studies. However, to allow direct comparison, we used the full dataset of the 2006 cohort as the baseline while age-matched subsets of the 2008 and 2013 cohorts were used.

The population-averaged prevalence (referred to as prevalence) of *P. falciparum* and *P. vivax* infections in the three cohorts was estimated using generalised estimating equations (GEE) with a logit link and an exchangeable working correlation matrix, to account for the dependency between observations from the same child. Robust standard errors were also used to correct for working correlation matrix misspecification. Incidence rates (IR) for clinical episodes were calculated from the total number of clinical episodes experienced by each child over the study period and was modelled using negative binomial regression for the 2006 and 2013 cohorts and Poisson regression for the 2008 cohort. The relative percentage change in the prevalence and incidence was calculated using the formula: percentage change = ((current estimate − previous estimate)/previous estimate) × 100. Both the frequency of sampling and duration of blood-stage infections [51] are important factors influencing the _mol_FOB variable. Due to the differences in the frequency of sampling in the 2006, 2008 and 2013 cohorts, it was necessary to censor any sampling time-points that were not available across all three cohorts in order to be able to directly compare the _mol_FOB estimate across the cohorts. Incidence of new clones was defined as the sum of all new clones over the study period and derived using negative binomial regression, adjusting for individual time of exposure.

#### Analysis of key determinants of malaria infection and illness during the time of low transmission 2013

Risk factors of infection and malaria episode investigated in 2013 included the child’s age (years), timing of active detection of infection visits, area of residence, bednet use in the previous night, history of febrile illness in the past 2 weeks, presence of febrile illness, which is defined as the 2-day history of fever ± axillary temperature ≥ 37. 5 °C, and haemoglobin levels.

For all risk factor analyses, both univariable and multivariable regression models including all risk factors were examined. The association between prevalence of infections at monthly time-points and the risk factors was estimated using GEEs with a logit link and exchangeable working correlation matrix. Incidence of new blood-stage infections was estimated using GEE with negative binomial regression and an exchangeable working correlation matrix. Due to a very low number of clinical episodes observed in 2013, we used the total number of clinical episodes for each child across the follow-up period to assess the association between incidence of clinical infections and the risk factors. This was estimated using negative binomial regression. The risk factors were summarised across the study period for each child as follows: age at enrolment, residence (assumed not to vary across follow-up), mean haemoglobin level and _mol_FOB. Two multivariable models of the incidence of clinical infections, one including all aggregated risk factors and _mol_FOB (_mol_FOB-adjusted model) and the other excluding _mol_FOB (base model) were examined.

Due to reduced levels of transmission in 2013, several villages had few *P. falciparum* or *P. vivax* infections detected, no clinical *P. falciparum* or *P. vivax* episodes and very few new blood-stage clones. Therefore, villages were grouped into 4 areas with geographically similar characteristics (1 = Ilahita 1, 2, 3, 4, 6 and 7; 2 = Balanga and Balif; 3 = Kamanokor and Ilahita 5; and 4 = Sunuhu 1 and 2). Due to the universally high bednet use, analyses of their association with incidence of new blood-stage infections and clinical episodes did not converge and bednet use was excluded from both analyses. The associations are expressed as odds ratio (OR) and incidence rate ratios (IRR) and were considered to be statistically significant if the Wald test *p* value was below the nominal level of significance of 0.05.

The analyses were conducted using Stata 12.0 (StataCorp, USA) and R v2.12 (2011) [2006 cohort _mol_FOB analysis] and v3.4.0 (2017) [2008 cohort analyses] (R Core Team, R: A language and environment for statistical computing. R Foundation for Statistical Computing, Vienna, Austria).

## Results

### Changing burden of malaria infections and illness: 2006–2013

The prevalence of infection, _mol_FOB and incidence of clinical malaria were compared across three independent age-matched child cohorts conducted before (cohort 1, *n* = 264) and during (cohort 2, *n* = 149; cohort 3, *n* = 371) the intensification of malaria control activities. The overall prevalence of all *Plasmodium* spp. infections by PCR was 79.4% (CI_95_ 76.7–81.9%) in 2006, 77.0% (CI_95_ 73.4–80.3%) in 2008 and 25.6% (CI_95_ 22.5–29.0%) in 2013, with *P. vivax* the predominant species across all time-points.

**Fig. 2:**
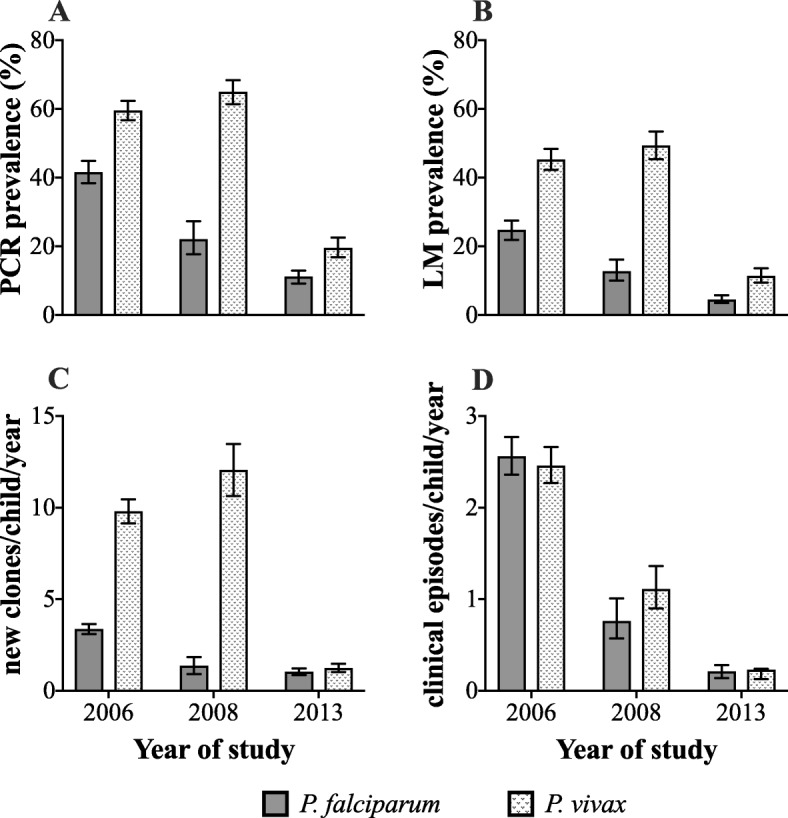
Changing burden of malaria infections and illness across the different time-points of malaria control intensification in the study area. Legend: Impact of improved malaria control on prevalence of infections detectable by **a** polymerase chain reaction assay (PCR), **b** light microscopy (LM), **c** incidence of new blood-stage infections (_mol_FOB) and **d** incidence of clinical malaria episodes. Error bars are 95% confidence intervals

In 2006, 2 years prior to the scale-up of control activities in the study area, prevalence of *P. falciparum* and *P. vivax* was 41.6% (CI_95_ 38.4–44.9%) and 59.6% (CI_95_ 56.6–62.4%) by PCR and 24.8% (CI_95_ 21.9–27.6%) and 45.3% (CI_95_ 42.3–48.3%) by LM, respectively (Fig. 2a, b). Two years later and within several months of the first population-wide distribution of LLIN by the National Malaria Control Program, the prevalence of *P. falciparum* almost halved [PCR 22.1% (CI_95_ 7.7–27.3%); LM 12.8% (CI_95_ 10.0–16.2%)), Fig. 2a, b], with little observed impact on *P. vivax* prevalence [PCR 65.0% (CI_95_ 61.4–68.4%); LM 49.4% (CI_95_ 45.4–53.5%), Fig. 2a, b]. However, after 5 years of sustained control in the area, the prevalence of *P. vivax* had also substantially declined (PCR 19.6% (CI_95_ 16.9–22.6%); LM 11.4% (CI_95_ 9.5–13.6%), Fig. 2a, b), and *P. falciparum* prevalence had continued to decline further to 11.2% (CI_95_ 9.2–13.0%) by PCR and 4.5% (CI_95_ 3.5–5.8%) by LM in 2013 (Fig. 2a, b). Infections due to *P. malariae* [2006 (7.9%), 2008 (4.1%), 2013 (0.3%)] and *P. ovale* [2006 (3.5%), 2008 (3.0%), 2013 (0.2%)] were only occasionally detected by PCR and also declined from 2006 to 2013.

As observed with the prevalence of infections, the incidence of *P. falciparum* genetically distinct blood-stage infections substantially declined following the first LLIN distribution. In contrast, *P. vivax*_mol_FOB did not change over the same interval. *P. falciparum*_mol_FOB decreased from 3.4 clones/child/year-at-risk (CI_95_ 3.1–3.6) in 2006 to 1.4 clones/child/year-at-risk (CI_95_ 0.9–1.8) in 2008, which further declined to 1.0 clones/child/year-at-risk (CI_95_ 0.9–1.2) in 2013 (Fig. 2c). In contrast, *P. vivax*_mol_FOB was observed to increase from 9.8 clones/child/year-at-risk (CI_95_ 9.1–10.5) in 2006 to 12.1 clones/child/year-at-risk (CI_95_ 10.6–13.5) in 2008, before declining to 1.2 clones/child/year-at-risk (CI_95_ 1.0–1.5%) in 2013 (Fig. 2c).

Interestingly, a different pattern was observed for the incidence of clinical *P. vivax* episodes (Fig. 2d). In spite of the persistence of a relatively high *P. vivax* prevalence and _mol_FOB following the first LLIN distribution, the incidence of clinical *P. vivax* declined by 55% in 2008 (2006, 2.46 episodes/child/year-at-risk (CI_95_ 2.27–2.66); 2008, 1.11 episodes/child/year-at-risk (CI_95_ 0.90–1.36)), before further declining to 0.23 episodes/child/year-at-risk (CI_95_ 0.13–0.24) in 2013. This corresponded to an overall reduction of 91% between 2006 and 2013. The incidence of clinical *P. falciparum* exhibited a similar pattern to that of the prevalence and _mol_FOB, with a continuous decline (2006, 2.56 episodes/child/year-at-risk (CI_95_ 2.36–2.77); 2008, 0.76 episodes/child/year-at-risk (CI_95_ 0.57–1.01); 2013, 0.21 episodes/child/year-at-risk (CI_95_ 0.14–0.28)), corresponding to an overall reduction of 92% between 2006 and 2013 (Fig. 2d).

### Key determinants of malaria infection and illness during the time of low transmission 2013

#### Demographic characteristics of enrolled participants

Of the 465 children enrolled into the 2013 cohort, data from 420 were available for analyses (retention rate 90%). These children ranged in age from 0.9 to 6.4 years (mean 3.3), 53.8% were male and 93% reported sleeping under a bednet the previous night. On average, the children attended 8 out of the 10 [range 1–10] active detection of infection visits.

#### Prevalence of infections during follow-up

Throughout the follow-up period, 47% children had at least one *P. falciparum* infection and 48% had at least one *P. vivax* infection (detected by PCR). Overall, the averaged prevalence of *P. vivax* was 19.9% by PCR and 10.8% by LM, while *P. falciparum* prevalence was 11.0% by PCR and 4.2% by LM. Sub-microscopic infections accounted for 64% of *P. falciparum* and 47% of *P. vivax* infections.

The prevalence of PCR-detectable infections varied markedly across the different areas (*Pf*: range 4.5–28.8%, *Pv*: range 6.0–45.2%; Table 1) with significantly higher risk of infection observed amongst children living in Sunuhu 1 and 2 compared to Ilahita 1, 2, 3, 4, 6 and 7 (*Pf* crude OR 8.49 (CI_95_ 6.14–11.8) *p* < 0.001, *Pv* 12.6 (CI_95_ 8.11–19.6) *p* value < 0.001); Additional file 1). Whereas the prevalence and the risk of *P. falciparum* infections also varied significantly over time (range 7.1–32.2%, *p* < 0.0001), *P. vivax* prevalence and risk was more stable over time (range 17.8–23.2%, *p* = 0.1777; see Table 1 and Additional file 1). The risk of both *P. falciparum* and *P. vivax* infections was higher in children experiencing a febrile illness in the last 2 weeks (*Pf*: crude OR 2.97 (CI_95_ 1.57–5.63) *p* = 0.001, *Pv* 1.68 (CI_95_ 1.06–2.66) *p* = 0.028), as well as those with an enlarged spleen (*Pf*: crude OR 2.25 (CI_95_ 1.23–4.11) *p* = 0.009, *Pv* 1.82 (CI_95_ 1.07–3.11) *p* = 0.028); see Additional file 1). The prevalence and risk of *P. falciparum* infections was also increased in children experiencing a concurrent febrile illness (crude OR 2.28 (CI_95_ 1.66–3.15) *p* = 0.001), increased linearly with age (crude OR 1.24 (CI_95_ 1.09–1.41) *p* = 0.001) but declined for every 1 g/dL increase in haemoglobin level (crude OR 0.72 (CI_95_ 0.64–0.80) *p* < 0.001; Additional file 1). Bednet use was associated with a reduced prevalence of infections for both species (*Pf* crude OR 0.58 (CI_95_ 0.27–1.29) *p* = 0.182, *Pv* 0.80 (CI_95_ 0.45–1.40) *p* = 0.431), but the very low number of non-users results in insufficient power. Having received recent antimalarial treatment was associated with a decrease in *P. vivax* (crude OR 0.36 (CI_95_ 0.15–0.85) *p* = 0.021; Additional file 1) prevalence and risk.

**Table 1:** Key predictors of infections due to *P. falciparum* and *P. vivax* as detected by qPCR in 2013

	*P. falciparum*				*P. vivax*			
	Observed positive (%; *n* = 4363)	OR	CI_95_	*p*	Observed positive (%; *n* = 4363)	OR	CI_95_	*p*
Areas of residence								
Ilahita 1–4, 6, 7	4.5	Reference group			6.1	Reference group		
Balanga and Balif	4.6	1.01	0.59–1.72	0.969	6.0	0.98	0.51–1.88	0.946
Kamanokor and Ilahita 5	12.9	2.29	1.38–3.80	0.001	38.0	9.22	5.55–15.3	< 0.001
Sunuhu 1 and 2	28.8	7.63	5.34–10.9	< 0.001	45.2	13.7	8.81–21.3	< 0.001
			*p* < 0.0001^a^				*p* < 0.0001^a^	
Age								
Linear		2.91	1.26–6.70	0.012		1.32	1.16–1.51	< 0.001
Quadratic		0.88	0.78–0.99	0.032				
ADI visit								
Enrolment	18.4	Reference group			23.2	Reference group		
Week 4	8.7	0.40	0.27–0.58	< 0.001	21.4	0.80	0.61–1.05	0.105
Week 8	8.7	0.39	0.19–0.46	< 0.001	21.3	0.85	0.65–1.11	0.238
Week 12	7.1	0.30	0.19–0.46	< 0.001	19.1	0.71	0.53–0.96	0.024
Week 16	8.1	0.34	0.22–0.53	< 0.001	17.8	0.65	0.49–0.88	0.004
Week 20	9.9	0.47	0.27–0.79	0.004	20.3	0.66	0.45–0.98	0.04
Week 24	7.6	0.31	0.19–0.52	< 0.001	21.8	0.83	0.59–1.19	0.312
Week 28	7.2	0.36	0.22–0.60	< 0.001	17.9	0.64	0.43–0.94	0.024
Week 32	8.8	0.40	0.25–0.65	< 0.001	18.1	0.57	0.40–0.83	0.004
Week 36	10.8	0.55	0.35–0.85	0.007	18.0	0.53	0.37–0.76	0.001
Week 40	32.2	3.20	2.15–4.74	< 0.001	22.9	0.82	0.56–1.19	0.298
			*p* < 0.0001^a^				*p* = 0.0129^a^	
Haemoglobin		0.65	0.57–0.74	< 0.001				
Recent antimalarial								
No	11.5				20.3			
Yes	21.3				15.0	0.34	0.17–0.71	0.004
Enlarged spleen								
No	10.8				19.2			
Yes	38.6				54.6	1.66	0.98–2.79	0.059
Febrile illness								
No	10.9				19.7			
Yes	25.0	1.84	1.30–2.62	0.001	29.2			
^b^2 weeks history of febrile illness								
No	11.5				20.0			
Yes	28.6	2.24	0.93–5.38	0.073	38.1	1.84	1.02–3.32	0.042

Multivariate GEE model-based estimates of risk of infection detected at each monthly active case detection visit time-point via backward selection of significant risk factors. *OR* multivariate adjusted odds ratio, *CI*_*95*_ 95% confidence interval, *p p* value, *ADI* active detection of infection. ^a^Overall significance level for the variable estimated using Wald chi-square test. ^b^Excluding febrile illness at the time of visit

In multivariate analyses, area of residence, time of visit, age, haemoglobin level and the presence of a concurrent febrile illness remained independently associated with the presence of a *P. falciparum* infection (Table 1). Area of residence, time of visit, recent antimalarial use, age and having an episode of febrile illness in the previous 2 weeks were all associated with the risk of carrying a *P. vivax* infection (Table 1). Risk factors of LM-detectable infections were similar (see Additional file 2).

#### Molecular force of blood-stage infections in monthly intervals

Incidence of new blood-stage infections was determined for a total of 303.4 person-years of follow-up with each child at risk of acquiring new blood-stage infections for an average of 0.73 years during the cohort. The mean _mol_FOB for *P. falciparum* was 1.6 (CI_95_ 1.4–1.9) new infections per child per year-at-risk and 2.2 (CI_95_ 1.9–2.6) infections/child/year-at-risk for *P. vivax*.

The rate of acquiring new *P. falciparum* clones was higher in Sunuhu 1 and 2 compared to Ilahita 1, 2, 3, 4, 6 and 7 (*Pf* IRR 3.10 (CI_95_ 2.08–4.63) *p* value < 0.001) and also in those with recent antimalarial use (IRR 10.4 (CI_95_ 5.92–18.2) *p* value < 0.001, Table 2). Age was not associated with *P. falciparum*_mol_FOB in multivariate analysis despite the significant linear association observed in the crude analysis. The *P. vivax*_mol_FOB was increased in both Sunuhu 1 and 2 and Kamanokor and Ilahita 5 compared to Ilahita 1, 2, 3, 4, 6 and 7 (IRR 8.16 (CI_95_ 5.38–12.4) *p* value < 0.001 and 6.66 (CI_95_ 4.24–10.5) *p* value < 0.001, respectively), and also increased linearly with age (IRR 1.26 (CI_95_ 1.13–1.40) *p* value < 0.001, Table 2). Both *P. falciparum* and *P. vivax* incidence varied markedly over the follow-up time period (both *p* < 0.0001, Table 2).

**Table 2:** Multivariate predictors of molecularly determined new *P. falciparum* and *P. vivax* blood-stage infections in 2013

	*P. falciparum*				*P. vivax*			
	IR	IRR	CI_95_	p	IR	IRR	CI_95_	p
Areas of residence								
Ilahita 1–4, 6,7	1.09	Reference group			0.65	Reference group		
Balanga and Balif	1.25	1.22	0.70–2.12	0.485	0.94	1.46	0.81–2.64	0.213
Kamanokor and Ilahita 5	1.82	1.61	0.92–2.80	0.096	4.83	6.66	4.24–10.5	< 0.001
Sunuhu 1 and 2	3.57	3.10	2.08–4.63	< 0.001	5.37	8.16	5.38–12.4	< 0.001
			*p* < 0.0001^a^				*p* < 0.0001^a^	
Age						1.26	1.13–1.40	< 0.001
ADI visit interval								
Enrolment–week 4	1.23	Reference group			3.20	Reference group		
Week 4–week 8	0.85	0.58	0.27–1.23	0.153	2.62	0.71	0.52–0.98	0.035
Week 8–week 12	0.22	0.15	0.06–0.38	< 0.001	1.60	0.44	0.30–0.63	< 0.001
Week 12–week 16	0.77	0.50	0.23–1.09	0.081	2.30	0.59	0.43–0.82	< 0.001
Week 16–week 20	1.23				3.20	0.87	0.60–1.25	0.446
Week 20–week 24	2.58	1.99	1.00–3.96	0.049	3.60			
Week 24–week 28	1.17	0.83	0.43–1.61	0.583	2.20	0.57	0.41–0.79	< 0.001
Week 28–week 32	1.13	0.84	0.43–1.64	0.602	1.98	0.48	0.34–0.69	< 0.001
Week 32–week 36	1.28	0.89	0.49–1.64	0.719	2.46	0.58	0.42–0.80	< 0.001
Week 36–week 40	7.19	5.55	3.33–9.25	< 0.001	2.26	0.56	0.39–0.79	< 0.001
			*p* < 0.0001^a^				*p* < 0.0001^a^	
Recent antimalarial use^b^	8.50	10.4	5.92–18.2	< 0.001	2.79			
Febrile illness	2.05				3.02			
2 weeks history of febrile illness^c^	2.11				2.31			
Haemoglobin								
≥ 10 g/dL	1.60				2.15			
9–9.9 g/dL	1.88				2.48			
≤ 9 g/dL	2.06				2.87			

Estimates from a multivariate negative binomial regression with GEE model predicting risk of acquiring new species-specific clones for *P. falciparum* and *P. vivax* in a 4-week interval when the child was considered at risk. A backward selection approach was used with the best fitting model consisting of the significant associations. *IR* incidence rate, *IRR* incidence rate ratio, *CI*_*95*_ 95% confidence interval, *p p* value, *g/dL* grams/decilitre, *ADI* active detection of infections. ^a^Overall significance level for the variable estimated using Wald chi-square test. ^b^Antimalarial treatment within 28 days before the start of the interval. ^c^Excluding febrile illness at the time of visit

#### Predictors of clinical malaria episodes

Over the 10 months of follow-up, a total of 366 febrile illness episodes were observed, of which 109 (30%) were associated with microscopically confirmed infections (IR, 0.36/child/year), with 51 *P. vivax* (any density: IR, 0.19) and 49 *P. falciparum* (any density: IR, 0.18) episodes. Another 7 were *P. falciparum* and *P. vivax* mixed infections (any density: IR 0.02), 2 were *P. malariae* (any density: IR, 0.07). Clinical episodes with high-density parasitaemia (≥ 2500 for *P. falciparum* and ≥ 500 for *non-falciparum* infections) accounted for 63.3% (35 *Pf*, 27 *Pv*, 7 *PfPv* mixed) of all the clinical episodes. There were no *P. ovale* clinical episodes observed.

The incidence of clinical *P. falciparum* episodes was significantly higher in Kamanokor, Ilahita 5 and Sunuhu 1/2 compared to Ilahita 1, 2, 3, 4, 6 and 7 (IRR 4.30 (CI_95_ 1.59–11.6) *p* value 0.004 and 8.15 (CI_95_ 3.40–19.6) *p* value < 0.001, respectively; Table 3). Each 1 g/dL increase in haemoglobin was associated with a 48% reduction in the incidence of clinical *P. falciparum* (CI_95_ 0.35–0.77, *p* value: 0.001, Table 3), and each 1-year increase in age was associated with a 38% increase in the rate of clinical *P. falciparum* (CI_95_ 1.10–1.73, *p* value: 0.006, Table 3). After adjustment for _mol_FOB, all remained associated with the rate of clinical *P. falciparum* episodes, and a unit increase in _mol_FOB (i.e. one new *P. falciparum* infection per child per year-at-risk) was associated with a 10% (CI_95_ 1.02–1.18, *p* value 0.008) increase in the rate of clinical *P. falciparum* infections (Table 3).

**Table 3:** Key predictors of clinical malaria episodes due to *P. falciparum* and *P. vivax* in 2013

	*P. falciparum*					*P. vivax*				
		Base model		_mol_FOB adjusted			Base model		_mol_FOB adjusted	
	IR	IRR (CI_95_)	p	IRR (CI_95_)	p	IR	IRR (CI_95_)	p	IRR (CI_95_)	p
Areas of residence										
Ilahita 1–4, 6, 7	0.05	Reference group				0.06	Reference group			
Balanga & Balif	0.03	0.63 (0.13–3.14)	0.575	0.56 (0.11–2.81)	0.485	0.06	1.15 (0.32–4.06)	0.833	1.08 (0.30–3.91)	0.909
Kamanokor & Ilahita 5	0.25	4.30 (1.59–11.6)	0.004	3.96 (1.46–10.8)	0.007	0.52	8.01 (3.23–19.9)	< 0.001	3.86 (1.44–10.3)	0.007
Sunuhu 1&2	0.50	8.15 (3.40–19.6)	< 0.001	6.48 (2.65–15.8)	< 0.001	0.33	3.71 (1.53–8.99)	0.004	2.00 (0.77–5.17)	0.152
		*p* < 0.0001^a^		*p* < 0.0001^a^			*p* < 0.0001^a^		*p* < 0.0234^a^	
Age		1.38 (1.10–1.73)	0.006	1.30 (1.03–1.64)	0.026					
Haemoglobin		0.52 (0.35–0.77)	0.001	0.61 (0.40–0.92)	0.017		0.31 (0.19–0.48)	< 0.001	0.38 (0.24–0.59)	< 0.001
FOB^b^				1.10 (1.02–1.18)	0.008				1.17 (1.09–1.25)	< 0.001

Multivariate negative binomial regression model-based estimates predicting risk of clinical *P. falciparum* and *P. vivax*. Backward selection approach was used to derive significant associations. Base models included all variables except _mol_FOB. Incidence is based on aggregated clinical data for entire 10-month study period thus precluding analysis of recent antimalarial treatment as a covariate. Bednet use was not analysed due to non-converge of data when included into models. ^a^Overall significance level for the variable estimated using wald chi-square test; _mol_FOB: molecular force of blood-stage infections; ^b^_mol_FOB was included as a rate; IR: Incidence rate; IRR: Incidence rate ratio. *CI*_*95*_ 95% confidence interval; *p p* value

The rate of clinical *P. vivax* episodes was also significantly higher in Kamanokor, Ilahita 5 and Sunuhu 1/2 compared to Ilahita 1, 2, 3, 4, 6 and 7 (IRR 8.01 (CI_95_ 3.23–19.9) *p* value < 0.001 and 3.71 (CI_95_ 1.53–8.99) *p* value 0.004, respectively; Table 3). Each 1 g/dL increase in haemoglobin was associated with a 69% reduction in the rate of clinical *P. vivax* (CI_95_ 0.19–0.48, *p* value < 0.001). After adjustment for _mol_FOB, only area of residence and haemoglobin remained associated with the rate of clinical *P. vivax* episodes (Table 3). A unit increase in _mol_FOB (i.e. one new *P. vivax* infection per child per year-at-risk) was associated with a 17% (CI_95_ 1.09–1.25, *p* value < 0.001) increase in the rate of clinical *P. vivax* infections. Age was not associated with the rate of clinical *P. vivax* episodes, either before or after adjustment for _mol_FOB.

## Discussion

This is the first study in a *P. falciparum/P. vivax* co-endemic area and amongst very few studies globally [52] to examine the impact of improved malaria control on the epidemiology of malaria in young children using longitudinal cohorts rather than the widely used nationwide and community household surveys and routine health information systems [6, 33, 37]. Longitudinal cohort studies allow for a detailed investigation into the dynamics of infection, and illness, as well as the rate of acquiring new infections (_mol_FOB) and clinical illness over time.

By analysing these metrics in three consecutive longitudinal cohorts in young PNG children, we demonstrate a differential impact of control interventions on *P. vivax* compared to *P. falciparum* that may be overlooked in routine surveillance. Following the first LLIN distribution, the prevalence of *P. falciparum* infection and both *P. falciparum* and *P. vivax* clinical episodes declined immediately and continuously across the time period of the three cohorts. Contrastingly, the prevalence and force of *P. vivax* blood-stage infections did not decline, remaining initially relatively high with a substantial decline only evident in the most recent cohort that was conducted 5 years after commencement of intensified control in the area. These observations confirm that key biological differences between the two species render them differentially susceptible to standard control tools such as LLINs and case management, highlighting the need for *P. vivax*-focused interventions in co-endemic regions.

Notably, the relationship between transmission and _mol_FOB differs for *P. falciparum* and *P. vivax*. *P. falciparum* metrics are directly linked to blood-stage infections, which are always mosquito-derived, hence closely reflecting current levels of transmission. The reductions in *P. falciparum*_mol_FOB observed across these three cohorts confirm reductions in *P. falciparum* prevalence and EIR observed through monitoring and evaluation of the national programme [37, 38]. Due to the biological ability of *P. vivax* to remain dormant in liver cells as hypnozoites and to serve as a continuing source of relapsing infections, *P. vivax* metrics are not able to differentiate between mosquito-derived and relapsing infections and therefore do not reflect active transmission as closely as *P. falciparum* metrics. This is particularly relevant in PNG, where *P. vivax* is the predominant species detectable in young children and relapses account for more than 50–80% of *P. vivax* infections in pre-school and primary school children [14, 41]. As a consequence, the *P. vivax*_mol_FOB is a composite measure reflecting the joint burden of new, mosquito-derived and relapsing infections [42, 43]. This metric therefore reveals a high burden of persisting low-density relapsing infections in young children, contrasting results of nationwide surveys that showed a comparable decline in *P. falciparum* and *P. vivax* prevalence detectable by LM in both children under 5 years and the general population [37].

Given the persistence of a high burden of *P. vivax* infections following the initial LLIN distribution, the observation that the burden of clinical *P. vivax* dropped and continued to decline over the years of intensification marked a striking difference. Clinical immunity to *P. vivax* is acquired rapidly, even under relatively low transmission [15]. In malaria therapy patients, only few mild febrile symptoms were observed when they were re-infected with a homologous infection [53]. As relapsing infections are either genetically identical or meiotic siblings of the primary infection [54, 55], it is generally thought that clinical episodes are more likely to be caused by new mosquito-bite-acquired infections. Considering that reduction in transmission results in the acquisition of fewer new mosquito-derived infections, the observation that the immediate impact of LLIN was exclusively on incidence of *P. vivax* clinical episodes and not on risk of infection strongly suggests that the majority of clinical episodes due to *P. vivax* may indeed be associated with mosquito-derived rather than relapsing infections.

The observation of a delayed impact of LLIN scale-up on *P. vivax* compared to *P. falciparum* blood-stage infections in co-endemic areas is important evidence for control programmes. It suggests that the large reservoir of hypnozoites acquired when transmission is high (prior to scale-up of control) gives rise to a sufficient burden of relapsing infections that may be transmissible, although often not symptomatic, such that minimal impact may be observed on *P. vivax* prevalence in the years immediately following scale-up even though transmission is being reduced. This highlights the importance of strengthening the implementation of radical cure of *P. vivax* in order to accelerate reduction in the burden of *P. vivax* [56]. Reluctance to prescribe primaquine without G6PD testing and poor adherence to the 14-day regime are major issues limiting the effectiveness of *P. vivax* radical cure in many settings, including PNG.

The observed impact on clinical incidence and the comparable longer-term reduction in *P. vivax* and *P. falciparum* burden of infections does however provide reassurance that vector control with LLINs can reduce the burden of *P*. *vivax*, at least in countries where malaria transmission is largely peri-domestic [57], even if coverage needs to be maintained for a longer period of time before the full effectiveness is observed. Interestingly, in many countries in Asia and the Americas where dramatic shifts to *P. vivax* predominance have been observed, programmes rely upon clinical case management (often with poor coverage of anti-hypnozoite therapy) as their primary malaria control strategy [2, 58] and/or have highly exophilic vectors with transmission occurring mainly in forested areas where LLIN and other traditional vector control tools such as indoor-residual spraying have limited efficacy [59–61].

During the period of reduced transmission in 2013, the individual level of exposure to new blood-stage infections (_mol_FOB) and the geographical location of the child’s residence were the two key determinants of infection and illness. In the previous 2006 and 2008 cohorts, age-dependent decreases in the incidence of clinical *P. vivax* were observed [40, 41], suggestive of rapid acquisition of clinical immunity due to high *P. vivax*_mol_FOB during those periods. Conversely, we did not observe any age association in 2013, which may be explained by the substantial decline in the force of *P. vivax* infection.

As documented in other settings, declining transmission leads to increasing transmission heterogeneity [60, 62] and an increasing proportion of asymptomatic low-density infections [6–8]. In 2013, over two thirds all PCR-detected infections were sub-microscopic and the risk of clinical malaria was highly dependent on where the child lived, with higher risk of clinical illness observed in areas with higher force of infection. This pronounced spatial heterogeneity in the risk of infections and malaria illness has also been observed in the two previous cohorts [40–43] indicating that despite the declining transmission between 2006 and 2013, the high burden areas remained stable. In particular, we observed marked geographical clustering of infections and illness in two areas, Sunuhu 1/2 and Kamanokor/Ilahita 5 in 2013, the same geographical locations that were identified as highest burden areas before [40, 42, 43] and during scale-up of interventions [41]. The persistence of high-burden areas such as these despite the ongoing implementation of control interventions is supported by observations made elsewhere [29, 30] and strengthens the rationale for surveillance strategies that target interventions to these potential transmission hotspots in order to accelerate control. Such strategies will clearly need to identify the characteristics of hotspots that fuel sustained transmission and address the diagnostic challenge imposed by asymptomatic, low-density infections [5, 63–65].

A limitation of this study is the differences in the study designs, sampling schedules and the length of follow-up as well as the non-uniform structuring of the individual datasets. Consequently, each cohort was analysed separately and the calculated burden of malaria infection and disease were compared between the cohorts to determine the patterns of decline for *P. falciparum* and *P. vivax* across the intervention time-points. As such, we did not statistically test the differential patterns of decline exhibited by *P. falciparum* and *P. vivax* across the intervention time-points. However, confidence intervals of the prevalence, _mol_FOB and clinical incidence across the three cohorts are provided illustrating when differences are statistically significant. It should also be noted that the cohorts were conducted in the same study area with a stable population and the cohorts were age-matched hence minimising variation between the cohorts.

Lastly, the impact of malaria control interventions on transmission are a function of diverse social and ecological settings leading to differences in mosquito abundance, mosquito behaviour and human-mosquito interaction. While improvements in the quality of housing have occurred over the past decade in many urban areas of PNG, housing for PNG’s rural majority remains largely dependent on bush material. Higher quality of housing and socio-economic status was associated with reduced risk of malaria in previous studies in PNG [37]; however, such data is not available from the child cohorts analysed. The association between weather patterns and long-term malaria trends in PNG have also been investigated using site-specific satellite weather variables and did not explain variations in observed malaria incidence over time (Rodríguez-Rodríguez & Hetzel, unpublished data). Further work understanding the dynamic, complex and responsive ecological niches driving ongoing malaria transmission in certain areas of PNG will inform the development of targeted control and elimination efforts.

## Conclusions

Scale-up of standard malaria control interventions in PNG substantially reduced the burden of malaria infection and disease in the most vulnerable 1–5-year-old age group. Data presented here suggests comparable reductions in new mosquito-derived infections for both *P. falciparum* and *P. vivax* but a delayed impact on *P. vivax* relapsing infections due to the previously acquired reservoir of hypnozoites. We confirm the effectiveness of sustained implementation of LLINs and case management in reducing transmission of both species in PNG but highlight the critical need to strengthen case management, radical cure, surveillance and targeted intervention strategies in order to accelerate control of malaria in co-endemic settings.

## Supplementary information

**Additional file 1:** **Table S1.** Bivariate associations between risk factors and the prevalence, _mol_FOB and clinical incidence. Estimates of bivariate associations calculated via generalised estimating equation (GEE) models for prevalence and molecular force of blood-stage (_mol_FOB) infections and negative binomial regression model used for clinical malaria episodes. Recent antimalarial use was not tested in the model for clinical malaria episodes due to aggregated clinical data. ^a^Age in years at enrolment was used for clinical malaria incidence while at the start of interval was used for _mol_FOB. ^b^Comparison group; For multilevel variables, comparison group estimates are presented as odds or incidence rate. PCR: Polymerase chain reaction assay; LM: light microscopy; OR: odds ratio; CI_95_: 95% confidence interval; IRR: incidence rate ratio.

**Additional file 2: Table S2.** Key predictors of the prevalence of infections due to *P. falciparum* and *P. vivax* as diagnosed by light microscopy. Multivariate generalised estimating equation (GEE) model-based estimates of the risk of infection detected at each monthly active detection of infection visits via backward selection of significant risk factors. ^a^Overall significance level for the variable estimated using wald chi-squared test. AOR: multivariate adjusted odds ratio. CI_95_: 95% confidence interval; ^b^Excluding febrile illness at the time of visit; Data for observed positive are %.

## Data Availability

Anonymised data is available upon reasonable request by contacting the PNG Medical Research Advisory Committee and the PNG Institute of Medical Research IRB. The contact is Dr. William Pomat, secretary PNGIMR IRB: William.Pomat@pngimr.org.pg.

## Abbreviations

AL: Artemether-lumefantrine
CI_95_: 95% confidence interval
DNA: Deoxyribonucleic acid
GEE: Generalised estimating equations
GPS: Global Positioning System
IR: Incidence rate
IRR: Incidence rate ratio
LLIN: Long-lasting insecticide treated nets
_mol_FOB: Molecular force of blood-stage infection
OR: Odds ratio
*p*: *p* value
PCR: Polymerase chain reaction
Pf: *P. falciparum*
PNG: Papua New Guinea
Pv: *P. vivax*
qPCR: Quantitative polymerase chain reaction
RDT: Rapid diagnostic test
spp.: Species
